# Does Failure to Register Matter? A Retrospective Comparison of Men on Probation for Sexually-Related Offenses

**DOI:** 10.1177/10790632261480681

**Published:** 2026-08-25

**Authors:** Leanne Fiftal Alarid, Adam K. Matz

**Affiliations:** 1 Department of Criminal Justice and Security Studies, 12337 University of Texas at El Paso, El Paso, TX, USA; 2 Department of Criminal Justice, College of Arts & Sciences, University of North Dakota, Grand Forks, ND, USA

**Keywords:** failure to register, probation, recidivism, revocation, sex offense

## Abstract

This study examined the extent to which men sentenced to probation for *Violation of Sex Offender Registration and Notification Act* (registration violations) differed from individuals sentenced directly to probation for a sex offense in a large urban county. All individuals in the study were revoked from probation over a three-year period. Variables of interest include actions that led to probation revocation, court-ordered condition violations that preceded revocation, court-ordered treatment requirements, criminal history, prior victimization, and other demographic characteristics. We found that the registration violation group was more likely to violate several conditions of probation, less likely to possess steady employment, and more likely to possess a history of prior charges including registration violations, drugs, theft and burglary, as compared to individuals directly sentenced to probation for a sex offense. Individuals in the registration violations group were also revoked with greater immediacy, within about 1.5 months on average compared to 3 months for individuals directly sentenced to probation. Probation officers may benefit from recognizing registration violation behaviors as distinctly different so that responses and strategies may be tailored to the greater likelihood of substance dependency, general criminality, and less cooperative tendencies.

## Introduction

The Jacob Wetterling Act of 1994 required persons convicted for felony sex offenses throughout the United States to register with law enforcement and mandated states to establish reporting standards.^1^ This act was later amended to allow public access to this information, and an international provision that shared sex offense conviction information inside US passports (McPherson, 2021).^2^ Registration timeframes ranged from 10 years to life, delineated by the nature of the offense, and victim’s age (Levenson et al., 2010). Public disclosure was initially assumed to increase safety by allowing citizens the chance to avoid known individuals, and to specifically deter these same individuals from reoffending. This assumption was based on state compliance with the law and continuously updated registration systems (Byre, 2022). Violations of the *Sex Offender Registration and Notification Act* (hereafter known as “registration violations”) became a separate felony crime if it was discovered that individuals with prior sex offense convictions failed to update their information in a timely manner (18 U.S.C. § 2250). These discoveries typically occurred during routine traffic stops or other circumstantial police contacts.

By 2006, the Adam Walsh Act mandated incarceration between one and 10 years for registration violations and required applicable individuals to update their information at least annually, according to Section 34 of the U.S. Code § 20913(f). This mandate significantly altered recidivism for new crimes committed by individuals with prior sex offense histories when rates were compared before versus after 2006. Prior to the Adam Walsh Act, 24% of individuals released from prison nationwide for a sex crime were reconvicted within 3 years for any new criminal offense; 5.3% arrested and 3.5% convicted for a new sex crime (Langan et al., 2003). Following Adam Walsh, registration violations were the most common offenses that contributed to reincarceration for individuals originally convicted of a sex offense. For example, registration violations in Minnesota (29%) were higher than committing a new sex offense (21%) for individuals released from prison (Duwe & Donnay, 2010). One additional study that compared earlier time periods before registration to individuals who were mandated to register after their release from prison for a sex offense found that the mandatory registration group was more likely to recidivate with drug-related offenses than the group released before registration (Jennings et al., 2014). While registration violations have contributed substantially to new prison admissions, judges began sentencing individuals to community supervision as an alternative to prison (i.e., probation) according to Cohen and Spidell (2016). Studies are virtually nonexistent on probation supervision outcomes for this group.

Recent figures estimate that 89,727 people in the United States were on probation and 83,856 were on post incarceration supervision (i.e., parole) for a sex offense at some time during the year (Kaeble, 2024). Individuals convicted of sex offenses are heavily scrutinized by the public, lack social support, and have a difficult time finding housing and employment (Kras, 2019; Levenson & Cotter, 2005; Levenson et al., 2007; Mercado et al., 2008; Tuschick et al., 2024) leading scholars to suggest that this high number of unintended collateral consequences may itself contribute to recidivism (Whittle, 2018). Court-ordered evaluations and treatment are added expenses, also creating a challenging situation for individuals without steady employment (Stalans, 2004). Probation and parole departments are under increasing pressure to improve how individuals convicted of sex offenses are supervised and treated, and at the same time, recognize early signs of recidivism (Kim et al., 2016; Stalans et al., 2004). The current study seeks to understand the role that registration violations have for individuals with a history of sex offenses in relation to other criminal behaviors and failures to comply with other court-ordered conditions that led to removal (i.e., probation revocation) and resentence to a treatment facility, jail, or prison. The term *supervision* is used interchangeably with the term *probation* to indicate individuals currently serving a sentence of supervised probation in the community (Alarid, 2019).

### Probation Supervision of Persons With Sex Offense Histories

Probation officers who supervise individuals convicted of sex offenses in the US commonly use the containment approach that includes specialized officer training, smaller case load size, frequent home visits, polygraph testing, mandatory treatment, and a close relationship with treatment providers (Cohen & Spidell, 2016; Drury et al., 2021; English et al., 1996; Pimentel & Muller, 2010). Home visits are conducted using teams of officers so that they may also check personal surroundings and computers to ensure appropriate internet use (Cumming & McGrath, 2005). In addition to conditions under the containment approach, individuals on probation must abide by standard probation conditions (typical across the US), to include regular reporting to the probation officer, remaining inside the jurisdiction, providing updated address or job change information, notifying probation officers of any police contacts, avoiding persons of disreputable character, refraining from possessing weapons, and abstaining from drugs and alcohol (Alarid, 2019).

Polygraph testing is a common tool used in probation supervision of individuals convicted of sex offenses in the United States (Pimentel & Muller, 2010) and United Kingdom (Kewley & Brereton, 2022). Uncovering potential violations of persons with sex offense convictions are believed by some to be more difficult due to the *appearance* of compliance through deception and a mastered ability to avoid detection (Payne & DeMichele, 2008). Polygraphs have been criticized for their lack of scientific validity (Collins, 2020; Meijer et al., 2008), yet they are widely used by treatment providers with justice-involved clients to detect deception during treatment sessions (Iacono, 2024). Polygraphs also help probation officers verify truthfulness as to whether probation conditions are being followed (no possession of pornographic material or obscene devices) and no commission of new sex offenses (Pimentel & Muller, 2010). The polygraph has had mixed results, from decreasing high-risk behavior (Grubin et al., 2004) to having no significant difference in sex recidivism rates between polygraphed and non-polygraphed individuals (McGrath et al., 2007).

While containment supervision has been widely used in probation settings throughout the U.S., only one empirical study could be located that found lower recidivism rates using containment supervision than when compared to standard probation (Stalans, 2004). Although the study was not able to pinpoint exactly which components of the containment approach were the most important, findings suggested that unannounced field visits and probation partnerships with treatment providers may offer two possibilities. Perhaps probation officers in specialized units were more likely to consider chronic criminal deviancy and extrafamilial victims when deciding whether to file a revocation (Stalans et al., 2004). All other studies found significant reductions in sex recidivism using containment when they examined individuals who first received treatment in prison before being released on parole or post-prison supervision (Aytes et al., 2001; Lowden et al., 2003; McGrath et al., 2003).

### Recidivism Correlates With Individuals on Probation

Previous studies have shown that sociodemographic factors significantly influence recidivism rates for individuals on probation for a sex offense (Freeman, 2007; Hepburn & Griffin, 2004; Kruttschnitt et al., 2000; Meloy, 2005). For example, prior criminal history (Freeman, 2007), being younger in age (Kruttschnitt et al., 2000; Meloy, 2005), non-White (Meloy, 2005) unemployed (Kruttschnitt et al., 2000), lacking residential and relationship stability (Hepburn & Griffin, 2004; Meloy, 2005), and having a history of drug and alcohol abuse (Hepburn & Griffin, 2004; Kruttschnitt et al., 2000; Meloy, 2005) all had a significant impact on likelihood of subsequent recidivism for individuals with convictions for sex offenses.

Two studies which are the most similar to the current study compared individuals on federal probation for a sex offense with individuals on probation for a registration violation for a previous sex offense (Cohen & Spidell, 2016; Drury et al., 2021). Cohen and Spidell (2016) used secondary data from 7,416 men who were placed on probation supervision for sex offenses between 2007–2013 throughout all 94 federal judicial districts in the US. They found that individuals on probation for registration violations (11.8% or *n* = 874) had a higher risk of recidivism score and were also more likely to be rearrested 3 years later (42%) than rearrest rates of individuals convicted of sexual assault (23%) or of child pornography possession (13%).

Drury and colleagues (2021) examined 216 individuals from a single federal probation district between 2016 and 2020. They compared a group of 38 individuals on probation for registration violations (17.5% of the sample) with all other individuals who were convicted of a sex offense, most commonly possession/distribution of child pornography. When compared with individuals on probation for a sex offense (*n* = 178), individuals supervised for registration violations (*n* = 38) had a more severe criminal history, to include an earlier age at initial involvement in criminal activity, accumulation of more arrests, spent more time in prison, and previously had more revocations on probation and/or parole. With regard to their current probation, individuals with registration violations were significantly more likely to be revoked for technical violations and 14 individuals (6.5%) out of 216 were revoked for committing a new crime. Of those 14 people, 5 were in the registration violation group (13% within this group) and 9 (5%) were on probation for sex offenses (Drury et al., 2021).

Researchers contend that probation supervision allows probation officers wide discretion to exercise differential enforcement practices, and this discretion may have an impact on recidivism rates (Thompson & Huebner, 2022). To reduce the number of technical violation cases that go before the judge, most probation departments allow the officers to use progressive sanctions which are written guidelines that direct officers in sanctioning court-ordered rule violations according to risk level and current offense. Progressive sanctions are not allowed when a new misdemeanor or felony crime is suspected, but progressive sanctions are strongly encouraged to provide individuals on probation more chances to change their behavior prior to formal court involvement. Hanson and Harris (2000) examined case file notes and interviewed probation officers about their views on technical violations. Probation officers reported that individuals who recidivated were uncooperative and disengaged with treatment, missed scheduled probation appointments, and showed more signs of anger, deception, and preoccupation with sexual deviance than individuals who succeeded on probation (Hanson & Harris, 2000). However, a literature gap exists on what is known about which other probation conditions within the containment approach are violated before a revocation decision is made.

### Failure to Register and Recidivism Using Publicly Accessible Databases

A third line of inquiry examined the relationship between failure to register and future recidivism using publicly available registration databases with formerly incarcerated individuals. These studies found that failure to register did *not* significantly predict future sex offending, leading many to conclude that system registration was independent of sex offending (Duwe & Donnay, 2010; Levenson et al., 2010, 2012; Zgoba & Levenson, 2012; Zgoba & Mitchell, 2023). Failure to register was more strongly correlated with general or non-sex crimes (Levenson et al., 2012), but the direction of that relationship is convoluted with mixed results. In a group of youth who were convicted of sex crimes, youth who were registered were more likely than nonregistered youth to be reconvicted of a nonperson offense (Letourneau & Armstrong, 2008). In a later study, Freedman (2012) tracked formerly incarcerated adults with prior sex offense histories up to 9 years. He found that those who failed to register (21.5% of the group) had a greater likelihood of new convictions by 64%, compared with individuals who were registration compliant, in the opposite direction of Letourneau and Armstrong (2008). Freedman’s findings for registered individuals were similar to the 65% recidivism rate for a different group of individuals who had non-sex convictions (Levenson et al., 2010). Individuals who had failed to register were more likely to be convicted again for another registration violation offense (Duwe & Donnay, 2010).

## The Present Study

This study has two purposes. First, it seeks to determine if significant differences exist between individuals that violated the sex offender registration statute (known as registration violations) and other individuals sentenced directly to probation for sex offenses (direct probation referrals) but who complied with registration requirements, similar to studies by Cohen and Spidell (2016) and Drury et al., (2021). Second, the research sought to identify what variables significantly influence the rate at which probation revocation occurs and if these rates differed between the two groups. Based on prior studies (Cohen & Spidell, 2016; Drury et al., 2021; Duwe & Donnay, 2010; Levenson et al., 2010; Zgoba & Levenson, 2012; Zgoba & Mitchell, 2023), we hypothesize that individuals on probation for registration violations will differ in terms of a greater proclivity for general offending when compared to direct probation referrals. We predict that the registration violations group will have more prominent criminal histories that extend well beyond sex offending, including a history of prior registration violations. As such, we also anticipate that individuals on probation for registration violations will be associated with more imminent revocations than direct probation referrals. We do not expect to see significant differences amongst these two groups in terms of employment, relationship status, or victim demographic characteristics.

## Method

### Procedure

Secondary data were collected from inactive case files stored by an adult probation department situated in an urban metropolitan county in Texas. All clients had originally been under probation supervision for a felony sex offense that required registration, and all clients had been revoked at some point within a 3-year period. A 3-year period was selected because client file data of revoked and inactive cases were stored for only 3 years before being destroyed. Files from individuals who had successfully completed probation were not available. The total number of available revoked case files was 192. Data were coded from hard copy files to an Excel spreadsheet via an encrypted laptop computer. All data were de-identified prior to being provided for research purposes. The study protocol was approved by the Institutional Review Board at the University of Texas at San Antonio.

Two subgroups were identified: the first group had been sentenced to probation for not complying with the continuous registration requirement on a previous sex offense, a behavior which itself was a low-level felony offense. The second group was directly sentenced to probation for a sex offense and in compliance with registration. Texas state law required that individuals on community supervision for a reportable sex offense (Texas Code of Criminal Procedure, Article 62.001- 62.203) be supervised under the containment approach (Texas Department of Criminal Justice-Parole Division, 2016). This program required that supervised individuals, at their own expense, complete a polygraph once per year and an initial evaluation to determine the frequency and duration of treatment. The initial evaluation included a clinical interview, intelligence test, psychological test, and victim interest according to standards set by the Council on Sex Offender Treatment (Dallas, 2009). If treatment was recommended, individuals engaged in 90 minutes of group therapy once per week, also at their own expense. Every individual had to be reassessed annually with an interview and polygraph to determine risk and continuation of treatment (Dallas, 2009). The probation department regularly used progressive sanctions to guide and direct officers in sanctioning for technical violations according to risk level, current offense, and violations committed. Progressive sanctions were not applicable for absconders or new crimes committed.

### Sample

Out of the total of 192 revocations, 24 files were excluded because the client absconded, had been deported, or referred back to their original jurisdiction, resulting in a final sample size of 168 individuals. The sample consisted entirely of men because no women were revoked during the 3-year period under study.^3^ The average age overall was 32.5 years (*SD* = 10.6). Most people were identified in the system as Latino at 63.1% (*n* = 106); Caucasians comprised 23.8% (*n* = 40), and African Americans 13.1% (*n* = 22) of the sample. The race/ethnic breakdown in the sample was similar to the larger community reported by the U.S. Census Bureau of 64.4% Latino, 23.6% Caucasian, 7% African-American, and 5% Asian/Pacific Islander (https://www.data.census.gov/). A small number of the sample group had stable employment (13.1%, *n* = 22), a high school degree or greater (36.9%, *n* = 62), or were married/cohabitating with a partner (37.5%, *n* = 63). About 15.5% was diagnosed with a mental health disorder (*n* = 26) which was consistent with individuals on probation nationwide (Van Deinse et al., 2023). In terms of the Static-99R risk assessment, 4.8% (*n* = 8) were classified as *well above average risk*, 36.9% (*n* = 62) as *above average risk*, 49.4% (*n* = 83) as *average risk*, and 8.9% (*n* = 15) *below average risk*. No individual within the sample was classified as *very low risk*. The mean recidivism risk for the overall sample was *average risk* (3.0, *SD* = 1.7).

Among the sample, their victims were predominantly female (89.9%, *n* = 151), and the victim’s average age was 12.6 years (*SD* = 6.3). Most of the victims were adolescents at 61.3% (*n* = 103), with another sizeable proportion representing preadolescents at 29.8% (*n* = 50). Few victims associated with this sample were adults at 8.9% (*n* = 15). Victim characteristics in the current study were also consistent with other research (Cale et al., 2014; King et al., 2019; Peugh & Belenko, 2001).

### Measures

#### Registration Violations

In Texas, failing to register or neglecting to update information annually is a fourth degree felony for individuals who were required to register for 10 years; a third degree felony for individuals required to register for the rest of their life; and the punishment is increased to the next highest degree if the individual has previously been convicted of registration violations (Attorney General of Texas, 2018; Texas Code of Criminal Procedure, Article 62.001- 62.203). This sample includes individuals placed on supervision for registration violations as their current conviction (44.6%, *n* = 75), and the direct probation referral group who complied with registration, but were under direct probation for a sex offense (55.4%, *n* = 93). A variable was created to distinguish these two groups with registration violations coded as “1” and direct probation referrals coded as “0.” This variable, along with days to revocation, served as the two dependent variables of interest in this study.

#### Days to Revocation

The number of days from initiation of court-mandated community supervision until revocation was tracked and reported. The count data ranged between 30 and 249 days (i.e., about 1–8 months). Revocations averaged 71.4 days (*SD* = 46.6). Common for count data, these data are positively skewed as reflected by a median of 57.6 and a reported skewness of 1.63. A kurtosis of 2.57 is also indicative of a slightly more platykurtic (i.e., flatter) distribution. About 75.0% (*n* = 126) of the revocations occurred in less than 90 days, 51.8% (*n* = 87) in less than 60 days, and 22.0% (*n* = 37) were reportedly revoked at the 30-day mark.

#### Static-99R Risk Score

Static-99R is an empirically derived actuarial risk assessment tool containing 10 items based on demographic information within the individual’s criminal history records. The authors hand coded Static-99R risk scores using the score sheet provided in the revised 2016 Static-99R coding rules manual (Phenix et al., 2017). Scores were based on 10 items present within the existing dataset. The risk score can range from −3 to 12 with classifications of *very low risk* (−3, −2), *below average risk* (−1, 0), *average risk* (1–3), *above average risk* (4, 5), and *well above average risk* (6 or higher).

#### Probation Revocation Type

Revocations included new crimes and technical violations. A series of independent variables were dummy coded with “1” indicating the presence of a failure type and “0” indicating the absence of the phenomenon for a given case. Four categories of new crimes (78.6%, *n* = 132) were documented which included registration violations (35.7%, *n* = 60), assault (14.9%, *n* = 25), drug possession (11.3%, *n* = 19), and property crime (16.7%, *n* = 28). The remaining revocations were attributed to technical violations (21.4%, *n* = 36) and are discussed in more detail in the following section. Relatedly, the number of motions to revoke that were filed prior to revocation was also reported. The average number of revocation motions was 1.9 (*SD* = 0.87).

#### Technical Violations

Numerous probation conditions were tracked and monitored. The probation conditions examined in this study include drug and alcohol abstinence (62.5%, *n* = 105), avoiding persons with criminal records and/or harmful or disreputable places (25.6%, *n* = 43), reporting to the probation officer as scheduled or requested (67.3%, *n* = 113), avoiding contraband and pornographic material (61.3%, *n* = 103), providing updates on address and employment changes (54.2%, *n* = 91), and notifying probation officer in the event of any police contact or arrest (28%, *n* = 47). These variables were dichotomously coded with “1” indicating at least one instance of the violation had occurred prior to revocation. Related to drug and alcohol abstinence, data on the presence of any positive urinalysis tests were also provided (33.3%, *n* = 56). This variable was also dichotomously coded with “1” indicating at least one positive test for alcohol or drugs was obtained prior to revocation.

#### Court-Ordered Treatment

It is well documented that treatment completion lowers recidivism for individuals with sex offense histories (Aytes et al., 2001; Gannon et al., 2019; Hanson et al., 2002; Holper et al., 2025). In Texas, the Council on Sex Offender Treatment sets all treatment standards and maintains a list of treatment providers throughout the whole state (Dallas, 2009). About 8 in 10 individuals (81.5%; *n* = 137) had been court-ordered to treatment for their most current sex offense (coded as 0 = no; 1 = yes). Most individuals not court-ordered to treatment were on probation for registration violations.

In a nationwide study, drug and alcohol use is common among two-thirds of individuals with histories of sex crimes (Peugh & Belenko, 2001) with alcohol abuse a predictor of recidivism (Looman & Abracen, 2011). Individuals ordered to drug and alcohol treatment (40.5%, *n* = 68) and/or for drug and alcohol abuse/dependency related to their offense (0 = no; 1 = yes) were collected.

#### Sanctions Prior to Current Sentence

Two variables captured the number of times an individual in the sample had been in state prison or placed on probation prior to their current probation. Each of these variables consisted of count data, representing a ratio-level of measurement. Overall, 51.8% (*n* = 87) of the sample had one or more prior stays in prison (*M* = 0.79, *SD* = 0.93) while 83.9% (*n* = 141) had previous experience being on probation (*M* = 2.52, *SD* = 1.95). The maximum number of times an individual had been to prison previously was four, with less than 12% of the sample having more than one such occurrence, compared to a maximum of 12 for probation sanctions, with just under 70% having been on probation two or more times.

#### Prior Criminal Charges

The influence of prior criminal behavior on subsequent offending behaviors is well documented (Hanson et al., 2003; Ozkan et al., 2020). Eleven variables indicated whether prior criminal charges were present, noting that these were arrests in which multiple charges were tied to an incident, and they were not necessarily convictions. A “1” indicated that this charge had been identified in an arrest before the current conviction, to include prior registration violations (17.3%, *n* = 29), sex-related offenses (17.9%, *n* = 30), misdemeanor drug charges (47.6%, *n* = 80), felony drug charges (26.2%, *n* = 44), driving while intoxicated (DWI) charges (23.8%, *n* = 40), theft and burglary charges (41.1%, *n* = 69), protective restraining order violation charges (6.0%, *n* = 10), assault charges (43.5%, *n* = 73), criminal mischief charges (13.7%, *n* = 23), weapons violation charges (22.6%, *n* = 38), and resisting or evading arrest charges (31.0%, *n* = 51).

#### Prior Victimization

Previous studies documented that many individuals convicted for sex offenses have themselves been previously abused as children (Cale et al., 2014; King et al., 2019; Peugh & Belenko, 2001), with significantly higher prevalence of sexual abuse history among individuals convicted of sex-related offenses compared to individuals convicted of non-sex-related offenses (Jespersen et al., 2009). In the present study, three dichotomously coded items were obtained that measured whether the individual on probation had been the victim of prior physical (17.3%, *n* = 29), emotional (14.3%, *n* = 24), or sexual abuse (6.0%, *n* = 10). These variables were asked during the initial evaluation and clinical assessment by a licensed professional, and provided to the probation department.

#### Demographics

Age at the start of probation (in years), and race/ethnicity were collected for individuals on probation. Race/ethnicity was dummy coded for three groups: African American, Caucasian, and Latino. Four additional variables were collected as protective factors and capabilities that individuals have to comply with probation requirements and/or change offending behaviors. These factors included if individuals on probation possessed steady employment (Kruttschnitt et al., 2000; Tuschick et al., 2024), 12 or more years of education (Stalans, 2004), were married or cohabitating with a partner (Kras, 2019), and whether they were diagnosed with a mental health disorder. Each of these variables were dichotomously coded with “1” indicating the presence and a “0” indicating the absence of the characteristic.

Victim gender (0 = male; 1 = female) and victim age (1–9 years = “preadolescent,” 10–17 years = “adolescent,” and 18 years and over = “adult”) were included due to victim characteristics being strong predictors of subsequent sex offending behaviors (Hanson et al., 2003; Stalans, 2004).

### Data Analysis

IBM SPSS Statistics (ver. 31) was used to conduct the data analysis. The authors take responsibility for the integrity of the data, the accuracy of the data analyses, and have made every effort to avoid inflating statistically significant results. Given that the analyses were exploratory with a sample size of 168, a relatively larger number of variables, and that the dataset represented only probation failures, the subsequent results focused primarily on descriptive statistics and bivariate analyses. For the bivariate analyses, the phi coefficient (*φ*) was performed when two variables of interest were each dichotomously coded (Ellis, 2010). Alternatively, the *Mann-Whitney U* test, a nonparametric alternative to the *t-test*, was utilized when examining associations between a dichotomous variable and any continuous variables (e.g., age, days), given these variables were often non-normal in their distributions (see Gibbons, 1993; Pett, 2016). A correlation coefficient (*r*) was computed and reported using the standardized test statistic (*z*), divided by the square root of the sample size (*n*). Finally, Spearman’s rho (ρ) was used for instances in which both variables of interest were continuous. To aid interpretation, it is recommended that an effect size (*r*, *φ*, ρ) of .10 should be considered small, .30 medium, and .50 a large effect (Ellis, 2010).

Table 1 presents descriptive data for the registration violation group separate from the direct probation referral group, with bivariate results provided in the right-most column to identify any statistically significant differences between the two groups. Table 2 provides further bivariate outputs using Days to Revocation as the dependent variable with the aim of identifying any statistically significant predictors correlated with the relative immediacy of revocation. These analyses were repeated for the overall sample and then again for registration violations and direct probation referrals separately.

**Table 1.** Bivariate Comparison of Individuals on Probation for Registration Violations and Direct Probation (*N* = 168)

Variable	Registration violations (*n* = 75)		Direct probation (*n* = 93)		*φ*/*r*
	*n*	%	*n*	%	
Plead guilty	41	54.7	44	47.3	.07
Static-99R risk score	*M* = 3.3	*SD* = 1.6	*M* = 2.8	*SD* = 1.7	.14
Probation failure type (revocation)					
New crime (while on probation)	63	84.0	69	74.2	.12
Registration violations only	26	34.7	34	36.6	−.02
Assault/Weapon	11	14.7	14	15.1	−.11
Drug possession	13	17.3	6	6.5	.20
Property/Mischief/DWLS	13	17.3	15	16.1	−.08
Technical violations only	12	16.0	24	25.8	−.12
Number of motions to revoke filed prior to revocation	*M* = 1.9	*SD* = 0.8	*M* = 1.9	*SD* = 0.9	.05
Days on probation prior to revocation	*M* = 49.2	*SD* = 29.5	*M* = 89.3	*SD* = 50.2	−.54^***^
Probation condition violations					
Abstain from drugs/Alcohol	52	69.3	53	57.0	.13
Positive UA drug/Alcohol test	28	37.3	28	30.1	.08
Avoid persons/Places of disreputable character	25	33.3	18	19.4	.16^*^
Report to probation officer	54	72.0	59	63.4	.09
No contraband, weapons, pornography, obscene devices	51	68.0	52	55.9	.12
Remain in jurisdiction/obtain permission to leave	19	25.3	22	23.7	.02
Provide updated address and report address/job changes	47	62.7	44	47.3	.15^*^
Notify officer of police contact/Arrest within 48 hours	27	36.0	20	21.5	.16^*^
Court-ordered treatment					
Treatment for sex offending	44	58.7	93	100.0	−.53^***^
Counseling for drug and alcohol abuse/Dependency	34	45.3	34	36.6	.09
Sanctions prior to current sentence					
Number of times in state prison	*M* = 1.5	*SD* = 0.8	*M* = 0.2	*SD* = 0.5	.77^***^
Number of times on probation	*M* = 3.3	*SD* = 1.9	*M* = 1.9	*SD* = 1.8	.41^***^
Prior criminal charges					
Registration violation(s)	20	26.7	9	9.7	.22^**^
Sex related offense(s)	12	16.0	18	19.4	−.04
Misdemeanor drug charge(s)	41	54.7	39	41.9	.13
Felony drug charge(s)	26	34.7	18	19.4	.17^*^
DWI charge(s)	23	30.7	17	18.3	.15
Theft and burglary charge(s)	39	52.0	30	32.3	.20^*^
Protective restraining order violation charge(s)	7	9.3	3	3.2	.13
Assault charge(s)	38	50.7	35	37.6	.13
Criminal mischief charge(s)	12	16.0	11	11.8	.06
Weapons violation Charge(s)	19	25.3	19	20.4	.06
Resisting/Evading arrest charge(s)	25	33.3	27	29.0	.05
Prior victimization					
If physically abused by someone else in the past	13	17.3	16	17.2	.01
If emotionally abused by someone else in the past	11	14.7	13	14.0	.01
If sexually abused by someone else in the past	6	8.0	4	4.3	.08
Sociodemographics of individuals on probation					
Age	*M* = 33.5	*SD* = 10.1	*M* = 31.6	*SD* = 10.9	.11
Race/Ethnicity					
African American	13	17.3	9	9.7	.11
Caucasian	16	21.3	24	25.8	−.05
Latino	46	61.4	60	64.5	−.03
Possess steady employment while on probation	5	6.7	17	18.3	−.17^*^
High school degree or greater	25	33.3	37	39.8	−.07
Married or cohabitating with a partner	25	33.3	38	40.9	−.08
Diagnosed with a mental health disorder	13	17.3	13	14.0	.05
Victim demographics					
Female	66	88.0	85	91.4	−.06
Age	*M* = 12.9	*SD* = 7.7	*M* = 12.4	*SD* = 4.8	−.05
Preadolescent (1–9 yrs)	27	36.0	23	24.7	.12
Adolescent (10–17 yrs)	40	53.3	63	67.7	−.15
Adult (18 yrs and over)	8	10.7	7	7.5	.06

*Note.* ^***^p < .001, ^**^p < .01, ^*^p < .05. Registration Violations coded as 1, Direct probation referrals as 0. Phi (*φ*) correlation is reported for all associations involving a categorical variable. *Mann-Whitney U*, a nonparametric alternative to the t-test, was utilized for all associations involving a continuous variable with *r* subsequently computed and reported for the effect size (*r* = z/√n). To aid interpretation, an effect size of .10 is considered small, .30 medium, and .50 large (Ellis, 2010). With exception to the victim’s age (*N* = 167), there was no missing data among these variables.

**Table 2.** Bivariate Comparison: Days to Revocation for Registration Violations and Direct Probation Groups (*N* = 168)

Variable	Overall (*N* = 168)	Registration violations (*n* = 75)	Direct probation (*n* = 93)
	*r/*ρ	*r/*ρ	*r/*ρ
Registration violations	−.54^***^	--	--
Plead guilty	−.08	−.04	−.07
Static-99R risk score	−.19^*^	−.18	−.12
Probation failure type (revocation)			
New crime (while on probation)	−.08	−.11	.08
Registration violations only	.11	.14	.11
Assault/Weapon	−.06	−.12	−.04
Drug possession	−.28^***^	−.26^*^	−.15
Property/Mischief/DWLS	.06	.09	.09
Technical violations only	.08	.11	−.08
Number of motions to revoke filed prior to revocation occurring	.29^***^	.32^**^	.41^***^
Probation condition violations			
Abstain from drugs/Alcohol	.05	.16	.12
Positive UA drug/Alcohol test	.05	.04	.14
Avoid persons/Places of disreputable character	−.09	.05	−.05
Report to probation officer	−.05	−.10	.08
No contraband, weapons, pornography, obscene devices	.01	−.04	.14
Remain in jurisdiction/obtain permission to leave	.02	.21	−.14
Provide updated address and report address/job changes	−.11	.01	−.04
Notify officer of police contact/Arrest within 48 hours	−.08	.05	−.04
Court-ordered treatment			
Treatment for sex offending	.64^***^	.80^***^	--
Counseling for drug and alcohol abuse/Dependency	.08	.27^*^	.02
Sanctions prior to current sentence			
Number of times in state prison	−.34^***^	.01	−.12
Number of times on probation	−.25^***^	−.16	−.08
Prior criminal charges			
Registration violations(s)	−.27^***^	−.21	−.10
Sex related offense(s)	.08	.17	.01
Misdemeanor drug charge(s)	−.03	.07	.04
Felony drug charge(s)	−.02	.00	.17
DWI charge(s)	−.03	.11	.00
Theft and burglary charge(s)	−.22^**^	−.07	−.14
Protective restraining order violation charge(s)	−.10	−.09	.09
Assault charge(s)	−.17^*^	−.16	−.04
Criminal mischief charge(s)	−.17^*^	−.17	−.14
Weapons violation charge(s)	−.17^*^	−.16	−.14
Resisting/Evading arrest charge(s)	−.15	−.02	−.24^*^
Prior victimization			
If physically abused by someone else in the past	.11	.07	.15
If emotionally abused by someone else in the past	.11	.09	.14
If sexually abused by someone else in the past	−.03	.03	−.06
Sociodemographics of individuals on probation	​	​	​
Age	−.03	.01	.07
Race/Ethnicity			
African American	.10	−.09	−.00
Caucasian	.05	.10	−.08
Latino	.03	−.02	.07
Possess steady employment while on probation	.19^*^	.12	.15
High school degree or greater	.19^*^	.13	.21^*^
Married or cohabitating with a partner	.18^*^	.14	.17
Diagnosed with a mental health disorder	.15	.00	−.25^*^
Victim demographics			
Female	.04	.14	−.13
Age	.05	−.05	.11
Preadolescent (1–9 yrs)	−.05	−.12	.05
Adolescent (10–17 yrs)	.04	.05	.05
Adult (18 yrs and over)	.01	.02	.05

*Note.* ^***^p < .001, ^**^p < .01, ^*^p < .05. Registration Violations coded as 1, Direct Probation Referrals as 0. *Mann-Whitney U*, a nonparametric alternative to the t-test, was utilized for all associations involving a categorical independent variable with *r* subsequently computed and reported for the effect size (*r* = z/√n). Spearman’s rho (ρ) was utilized for all associations reported with a continuous independent variable. To aid interpretation, an effect size of .10 is considered small, .30 medium, and .50 large (Ellis, 2010). With exception to the victim’s age (*N* = 167), there was no missing data among these variables.

Additional multivariate analyses were attempted with Poisson regression, a statistical procedure designed for when the dependent variable consists of count data (Weisburd et al., 2022). A key assumption of Poisson regression is that the conditional variance equals the conditional mean. If this is not true, the resulting model will be biased and misleading. These ratio measures have a true zero and each change in unit is of equal distance. Given count data are often positively skewed, skewness was already apparent when conducting bivariate analyses with this dataset, these procedures are more appropriate than ordinary least squares (OLS) regression which assumes normality and homoscedasticity of variance. The dependent variable of interest to these analyses is Days to Revocation from the start of probation. Recall that this sample includes only probation failures. Every case is associated with a specific time frame, counted in days. However, clear signs of over-dispersion were observed which necessitated using an alternative procedure: negative binomial regression. However, the negative binomial regression resulted in a non-significant model. Various combinations of independent variables, including subsets of key variables, were also attempted but no informative models could be produced. A lack of statistical power is evident by the study’s small sample size (Weisburd et al., 2022). Therefore, multivariate analyses are *not* reported with the subsequent results.

## Results

Table 1 provides descriptive and bivariate results comparing individuals on probation for registration violations (*n* = 75) and for direct probation referrals (*n* = 93) across several factors and covariates. Both groups appear to have similar Static-99R scores, prior sex offenses, and victim types of similar gender and age. Most notable differences were that the registration violation group was significantly more likely to have a history of prior registration violations compared to other individuals directly sentenced to probation for a sex offense (*φ* = .22, *p* = .004). This group was also more likely to have been in prison (*r* = .77, *p* < .001) and on probation (*r* = .41, *p* < .001), often multiple times, prior to the current sentence. Indeed, nearly all of the registration violation group had been in prison (96.0%, *n* = 72) or on probation previously (98.7%, *n* = 74). By contrast, the proportion of direct probation referrals with prior time in prison was relatively low at 16.1% (*n* = 15), though being on probation previously was similarly quite common at 72.0% (*n* = 67).

Though the effect sizes were relatively weak, individuals convicted of registration violations were less likely to comply with and subsequently possess more violations for several conditions of probation, including failing to notify the probation officer of a new arrest or police contact (*φ* = .16, *p* = .037), failing to provide updates on address and employment changes (*φ* = .15, *p* = .047), and associating with persons with criminal records or frequenting harmful places (*φ* = .16, *p* = .039). In addition, while steady employment was an issue for a majority of the sample overall, it was even more pronounced for the registration violation group at merely 6.7% compared to 18.3% for direct probation referrals (*φ* = −.17, *p* = .027). Finally, the registration violation group tended to have their supervision revoked sooner than other direct probation referrals, within about 1.5 months compared to 3 months for direct probation referrals (*r* = −.54, *p* < .001).

The extent to which the two groups experienced prior abuse themselves was not significantly different for physical abuse (17%) and for emotional abuse (14%), with 4% of direct probation referrals and 8% of the registration violation group experiencing previous sexual abuse. These rates were lower than rates reported in previous research (Cale et al., 2014; King et al., 2019; Peugh & Belenko, 2001). The difference in rates of childhood trauma is likely due to the data collection methodologies. In the current study, childhood abuse was divulged to a licensed psychologist, while most other studies distributed a research survey and used self-reported data (Jespersen et al., 2009). For the current offense, there were no significant differences between the two groups regarding the victim’s age as being a child or adult.

Further analyses were performed to identify specific factors associated with each probation group that increased or decreased the rapidness at which revocation occurred and whether these factors differed by group. The results can be found in Table 2. Bivariate analyses were repeated using the number of days from the start of probation to revocation as the dependent variable with the same set of independent variables in Table 1. Ten variables were found to have a statistically significant association with the number of days to revocation. Note that negative associations mean that higher scores on the measure were associated with revocation happening more quickly. These variables included if the current offense was for registration violations (*r* = −.54, *p* < .001), if the individual possessed a higher Static-99R risk score (*r* = −.19, *p* = .015), if revocation involved a new crime of drug possession (*r* = −.28, *p* < .001), number of times in prison prior to the current sentence (ρ = −.34, *p* < .001), number of times on probation previously (ρ = −.25, *p* < .001), if there was a history of prior registration violations (*r* = −.27, *p* < .001), a history of prior theft charges (*r* = −.22, *p* = .005), a history of prior assault charges (*r* = −.17, *p* = .033), a history of prior criminal mischief charges (*r* = −.17, *p* = .027), or prior weapons violations (*r* = −.17, *p* = .033), then a revocation tended to occur sooner than individuals who did not possess these characteristics. Alternatively, individuals with a greater number of motions leading up to revocation (*r* = .29, *p* < .001), if the individual had been ordered to participate in treatment (*r* = .64, *p* < .001), retained steady employment (*r* = .19, *p* = .013), earned the equivalent of a high school degree or greater (*r* = .19, *p* = .013), or was married (*r* = .18, *p* = .017) were all associated with a longer duration of probation supervision prior to revocation.

When repeating these analyses separately for each group, there was one consistent statistically significant variable across both groups: number of motions to revoke filed prior to revocation occurring. The association was stronger for individuals in the direct probation referrals group (ρ = .41, *p* < .001) compared to the registration violations group (ρ = .32, *p* = .005). Being court ordered to treatment for a sex offense was by far the strongest association (*r* = .80, *p* < .001) with days to revocation for the registration violation group. Recall that 58.7% of this group was court-ordered to treatment. The only other significant variables for the registration violation group were if mandated to counseling for drug and alcohol abuse (*r* = .27, *p* = .019), and if the new crime was for drug possession (*r* = −.26, *p* = .026). Three other variables were significant for direct probation referrals, but not for individuals on probation for registration violations: prior history of resisting or evading arrest charges (*r* = −.24, *p* = .022), if possessing a high school degree or greater (*r* = .21, *p* = .048), and if diagnosed with a mental disorder or illness (*r* = −.25, *p* = .017).

## Discussion

The present study sought to examine differences among a sample of individuals who had violated their probation. Individuals who were sentenced for registration violations on a previous index sex offense were compared to individuals directly sentenced to probation for an index sex offense. As expected, we found several significant differences between the two groups. First, as hypothesized, the registration violation group were more likely than the direct probation referral group to have a more extensive criminal history, more likely to have spent time in prison before, possess a history of prior registration violations, prior drug charges, as well as prior theft/burglary charges. Our findings here are consistent with prior probation research (Cohen & Spidell, 2016; Drury et al., 2021). Likewise, the registration violation group experienced new revocations more imminently than the direct probation referral group. There was no statistical difference observed between groups in terms of relationship status or victim demographics. However, contrary to our hypotheses, employment differed significantly between the two groups with the registration violation group less likely to maintain a steady job. Note, the registration violation group is likely comprised of individuals who initially went to prison for a sex offense, were released from prison, or completed their parole term. Given that these individuals were no longer under supervision, they failed to update their information in the database. They likely later encountered a police officer via a routine traffic stop or an employer who ran a background check and reported them, similar to Freeman’s (2012) findings. Given that Texas has indeterminate sentencing, judges used their discretion to sentence registration violations to probation.

This jurisdiction used progressive sanctions to extend time on probation, as evidenced by the number of motions leading up to revocation. While there were some individuals who had multiple chances to change their behavior prior to filing a revocation, it appeared that the revocation rate was significantly quicker for the registration violation group than for the direct probation referral group. The registration violation group in particular had more violations of standard probation conditions, which include failure to avoid persons of disreputable character, failure to provide updated address and employment information, and failure to notify the probation officer of law enforcement contact (all examples of standard probation conditions in numerous jurisdictions). These probation violation behaviors were consistent with other studies that found that individuals who had failed to register were more likely to be convicted again for another registration violation offense (Cohen & Spidell, 2016; Drury et al., 2021; Duwe & Donnay, 2010). Furthermore, in the present study, individuals on probation for registration violations were also less likely to possess steady employment, which also made it unlikely that they could afford to pay for treatment if court-ordered (Stalans, 2004).

Our findings suggest three possible explanations. First, that individuals who violated the registry for sex offenses were not necessarily a higher risk for sex offending, but some remained at a higher risk for general recidivism due to their more extensive criminal histories, and the likelihood of a higher risk client is less than for an individual in the direct probation referral group. Prior research is mixed in that registration violations are not associated with future sex offending in most research (e.g., Levenson et al., 2010; Levenson et al., 2012; Zgoba & Levenson, 2012; Zgoba & Mitchell, 2023). In contrast, other studies of individuals on federal probation supervision report a higher rate of sex offending (Cohen & Spidell, 2016; Drury et al., 2021). Individuals with registration violations have higher rates of general recidivism and further registration violations which is also consistent with extant research (Drury et al., 2021; Duwe & Donnay, 2010; Jennings et al., 2014). A second plausible explanation could be that for some men, returning to prison may feel like a less stressful option than the rigidity of lifetime registration and overwhelming number of probation conditions to follow (Tuschick et al., 2024). While our data did not include perspectives from individuals who were revoked, this perspective has been documented by others (Cubellis et al., 2019), and may apply to individuals in our study. Third, our findings may be indicative of inflexible probation policies for individuals convicted of registration violations, in a similar way that current registration and notification policies across the nation severely restrict housing choices, employment opportunities, entering school premises, and contribute to social ostracism (Byre, 2022; Tuschick et al., 2024).

Alternatively, present study findings included several protective measures that slowed the rate at which probation revocations occurred. The most notable variable was if the individual was court-ordered to treatment. In particular, there was a significant delay in revocation for the registration violation group who were mandated to treatment compared to those who did not have this mandate. This suggests that probation officers used a combination of discretion (Thompson & Huebner, 2022) and progressive sanctions to provide multiple chances for individuals to find full-time employment and to start treatment in an attempt to lower recidivism (Aytes et al., 2001; Gannon et al., 2019; Holper et al., 2025).

The presence of steady employment, sufficient educational attainment, and a strong marital/relationship status, to a small extent, all contributed to slowing down the rate at which individuals failed to complete their probation sentence, regardless of the type of offense in which the person was sentenced. This is largely as expected given that the impact of these variables has been established within the literature in relation to criminal behavior more broadly (Latessa et al., 2020). All three of these factors were supported by empirical research for individuals with sex offense histories as well (Kras, 2019; Kruttschnitt et al., 2000; Stalans, 2004; Tuschick et al., 2024).

From a practical standpoint, it may be beneficial for probation officers assigned to caseloads using the containment approach to be aware of the more general criminal propensities of individuals convicted for registration violations (Drury et al., 2021; Jennings et al., 2014) and recognize the need to identify, diagnose, and address problems including drug abuse (Hepburn & Griffin, 2004) and disreputable influences. The needs of this subpopulation appear to be more diverse. Further, it may be useful for probation officers to be aware of the lower likelihood of compliance with probation conditions compared to other individuals with prior sex offense histories (Drury et al., 2021). Advanced training on motivational interviewing may be of particular interest to officers to gauge motivation to make behavioral changes. Many justice-involved clients who are court-ordered to attend treatment, may be resistant or not fully participating in treatment on their own free will (Pinto e Silva et al., 2025; Westra & Norouzian, 2018).

Probation agencies may wish to consider a heightened level of supervision such as “intermediate supervision” (Center for Substance Abuse Treatment, 2005). This level of oversight may include increased drug testing, short jail stays, increased reporting to the probation officer, more frequent home visits, and additional requirements for attending relevant treatment and programming, particularly since outpatient treatment was found to be more effective than that offered in prison (Gannon et al., 2019; Holper et al., 2025; Kim et al., 2016). Emphasis should be on case planning and case management tailored to the needs of individuals within this population, supported by an actuarial risk/needs assessment tool (Corabian et al., 2010). Outpatient addiction treatment services may be a consideration with initial intensity that gradually tapers over the duration of the program as treatment goals are met. Likewise, the officer may reduce the frequency of check-ins and home visits as an incentive for supervision compliance more broadly. These outpatient programs may utilize a combination of cognitive-behavioral therapy, medication-assisted treatment, and therapeutic communities (Belenko et al., 2013; Corabian et al., 2010). The probation officer is a critical linkage point between the individual and the treatment provider. Effective referral and enrollment in addiction programming should not be understated (National Institute on Drug Abuse, 2018).

### Limitations

The current study relied on existing secondary data provided by a single jurisdiction and limits the extent of generalizability to other jurisdictions or types of justice supervision. The results do not apply to all groups of individuals convicted of a registerable sex offense. No data were available for similar individuals who successfully completed their supervision (estimated to be approximately 120 cases per year). This situation limited our comparison group and the extent of the potential analyses. The small sample size (i.e., low statistical power) also limited the analyses that could be conducted. There were 24 client files excluded for a variety of reasons that included the client absconded, had been deported, or referred back to their original jurisdiction. Another limitation of the current study concerned three specific variables. There were no women in the study, which did not allow for gender comparisons. The way that the race/ethnicity variable was collected by the agency did not account for individuals of mixed races. The time period since the last sex offense, although known for the direct probation group, was not known for the registry violators. Although time in the community without further criminal offenses has been associated with a reduction of risk (Hanson et al., 2024), the groups in the current study had high rates of criminal recidivism (84% of the registry violators and 74% of direct probation referrals), making it unlikely they are benefiting from any protective effect. A third limitation was that determining whether recidivism was the result of a subsequent registry violation or a new criminal behavior was inconclusive (Ostermann et al., 2020). Related to this is the possibility that filing a motion to revoke for technical violations is discretionary, and varies by probation officer (Thompson & Huebner, 2022).

That said, the current study does provide initial insights into unique characteristics that may be present with individuals with a registration violation conviction compared with individuals directly sentenced to probation for a sex offense. The evidence provided lends some credibility to the notion that there are circumstances and attributes that make this subpopulation unique. Alternative inpatient treatment strategies such as cognitive behavioral interventions for individuals convicted of sex offenses may be warranted before enforcing probation conditions. However, this research also calls into question the long-term efficacy of enforcing and sanctioning violations of registration without first providing treatment at the state’s expense (Dallas, 2009).

The future of continued database registration legislation is uncertain. Legal and academic analysts have called into question the deterrent value of registries (Socia & Stamatel, 2010; Wang, 2014). As of 2019, at least 34 states were unable to keep up with necessary registry database updates as required by law (Byre, 2022) leading some to speculate whether some parts of the legislation may be repealed altogether. Future research directions may include a 4-group longitudinal recidivism study comparing individuals who were successful on probation for a sex offense vs. registration violation, compared to individuals who were revoked on probation.

## Notes

1. Information that must be provided to US law enforcement includes: Full legal name, date of birth, social security number, phone number, email address, height, weight, eye color, hair color, shoe size, photograph, fingerprints, residential address, place of employment and/or school attended, offense of conviction, dates offense occurred, vehicle license plates, vehicle make/model, copies of passports, immigration documents, and professional licenses. Of this information, the general public has access to: Name, address, age, photo, height, weight, eye color, hair color, and conviction offense (34 U.S. Code § 20913). Each state has different time requirements for status updates. In Texas, residential address updates must occur within 7 days of moving, while other registration updates vary between 30-90 days depending on criminal history and the nature of the offense (Dallas, 2009).

2. Following the US creation of the registry for individuals convicted of sex offenses, a number of other countries have created their own registries for sex offense convictions, to include Australia, Canada, England, Ireland, New Zealand, Scotland, South Korea, and Wales (McPherson, 2021). To prevent the sexual exploitation of children during travel and tourism, these countries openly share information with international border agents and require applicable individuals to provide advance notification of foreign travel. Other countries that have created registries with more limited access include Argentina, Bahamas, Belize, Chile, Colombia, Cyprus, El Salvador, France, Guatemala, India, Jamaica, Japan, Kenya, Maldives, Nigeria, Pakistan, Paraguay, Peru, Poland, Portugal, Romania, Sierra Leone, Trinidad, Tobago, and Uruguay (McPherson, 2021).

3. Within this jurisdiction, approximately 350 people convicted of sex offenses or registration violations were on probation within that 3-year period and thus potentially revocable within the study’s time frame. Of the 350 people, only 5 were women.
